# The Hypopigmentation Mechanism of Tyrosinase Inhibitory Peptides Derived from Food Proteins: An Overview

**DOI:** 10.3390/molecules27092710

**Published:** 2022-04-22

**Authors:** Yuqiong Song, Shengjun Chen, Laihao Li, Yaoxun Zeng, Xiao Hu

**Affiliations:** 1 Key Laboratory of Aquatic Product Processing, Ministry of Agriculture and Rural Affairs, South China Sea Fisheries Research Institute, Chinese Academy of Fishery Sciences, Guangzhou 510300, China; syq1010730529@163.com (Y.S.); chenshengjun@scsfri.ac.cn (S.C.); laihaoli@163.com (L.L.); 2 Co-Innovation Center of Jiangsu Marine Bio-Industry Technology, Jiangsu Ocean University, Lianyungang 222005, China; 3 College of Food Science and Technology, Shanghai Ocean University, Shanghai 201306, China; 4 School of Biomedical and Pharmaceutical Sciences, Guangdong University of Technology, Guangzhou 510006, China; yx13630066913@126.com; 5 Collaborative Innovation Center of Provincial and Ministerial Co-Construction for Marine Food Deep Processing, Dalian Polytechnic University, Dalian 116034, China

**Keywords:** bioactive peptides, tyrosinase activity, hyperpigmentation, mechanism, molecular docking

## Abstract

Skin hyperpigmentation resulting from excessive tyrosinase expression has long been a problem for beauty lovers, which has not yet been completely solved. Although researchers are working on finding effective tyrosinase inhibitors, most of them are restricted, due to cell mutation and cytotoxicity. Therefore, functional foods are developing rapidly for their good biocompatibility. Food-derived peptides have been proven to display excellent anti-tyrosinase activity, and the mechanisms involved mainly include inhibition of oxidation, occupation of tyrosinase’s bioactive site and regulation of related gene expression. For anti-oxidation, peptides can interrupt the oxidative reactions catalyzed by tyrosinase or activate an enzyme system, including SOD, CAT, and GSH-Px to scavenge free radicals that stimulate tyrosinase. In addition, researchers predict that peptides probably occupy the site of the substrate by chelating with copper ions or combining with surrounding amino acid residues, ultimately inhibiting the catalytic activity of tyrosinase. More importantly, peptides reduce the tyrosinase expression content, primarily through the cAMP/PKA/CREB pathway, with PI3K/AKT/GSK3β, MEK/ERK/MITF and p38 MAPK/CREB/MITF as side pathways. The objective of this overview is to recap three main mechanisms for peptides to inhibit tyrosinase and the emerging bioinformatic technologies used in developing new inhibitors.

## 1. Introduction

Tyrosinase (TYR) is a metalloenzyme with a highly conserved copper binding region and exists in fruits, fungi, vegetables, mammals, cuticle sclerosis and wound healing in insects [1,2,3,4]. Two copper ions are essential for the catalytic activity of tyrosinase regardless of source [3]. In mammals, melanin regulated by tyrosinase is responsible for pigmentation of skin, hair and eyes [5]. When skin is exposed to UV radiation or oxidative stress, the melanocytes produce melanin, which accumulates in melanosomes, and is then transported to keratinocytes surrounding the melanocytes through dendrites to form supranuclear melanin caps to protect skin from photoaging [6]. Therefore, melanin is generally considered the perfect protection against UV damage. However, melanin is also the main reason for skin disorders, such as age spots, freckles and malignant melanoma [7].

Tyrosinase activity determines the synthesis content of melanin. Hence, inhibiting tyrosinase is one of the most effective ways to solve excessive pigment deposition [8]. At present, a large number of natural ingredients have been found to inhibit tyrosinase, among which phenols (flavonoids are the main ingredient), organic acids, glycosides, terpenes, aldehydes, esters, coumarins and their derivatives have better effects [9,10]. Kojic acid, hydroquinone and arbutin are mostly used in the treatment of melanin dermatosis. Although they have a strong inhibitory effect on tyrosinase activity, they are limited because of poor penetration and potential mutagenicity [11]. Finding inhibitors with high activity and low side effects has practical value in the prevention, or early treatment, of pigmented skin diseases [12].

As a new type of therapeutic drug, bioactive peptides have been of immense interest in recent years. Food-derived tyrosinase inhibitory peptides (TIPs) are favored, due to their high biological safety and easy absorption [13,14]. In addition to TIPs, amino acids released by gastrointestinal digestion can be absolutely absorbed without consumption. Active TIPs have been obtained from a wide range of animal and plant sources, and further animal and clinical trials are ongoing [15,16,17].

The present overview focuses on the sources, preparation, and inhibitory mechanisms on tyrosinase of TIPs and the emerging bioinformatic technologies used in studying TIPs, aiming to provide a theoretical basis and scientific guidance for dietary nutrition and cosmetics.

## 2. The Origin of Anti-Tyrosinase Peptides from Food Proteins

TIPs are short sequences including 3–20 units of amino acids obtained by enzymatic or chemical hydrolysis. Numerous studies reported that the anti-tyrosinase effect of TIPs is equivalent to, or even better than, that of natural or chemical synthetic inhibitors [18,19,20]. TIPs come from terrestrial and aquatic sources, and they are abundantly found in mammals’ milk [21] and agricultural products [22], as well as in aquatic products. In order to reduce costs, food industry by-products, such as peels [23], seeds [15], feathers [24], fish scales [25] and fish skin [26], are also utilized to produce potential TIPs.

### 2.1. Anti-Tyrosinase Peptides of Terrestrial Origin

TIPs play an important physiological role in organisms whose molecular weights are usually less than 6000 Da. The advantages of food protein hydrolysates include improved solubility, thermal stability and strong anti-precipitation ability [24]. Plant protein is a good material to obtain TIPs. The oldest study on natural TIPs isolated from Agaricus hortensis was reported by Madhosingh and Sundberg [27]. TIPs derived from land plants are usually found in crops, such as potato [22], rice [28], quince [15], and so on. These materials have also been utilized to cure other skin problems like inflammation and photoaging [29]. Peptide P4 (YRSRKSSWP) was known as one of the most effective anti-tyrosinase inhibitors (IC_50_ = 123 μmol/mL), and Ochiai et al. [30] found peptide TH10 (MRSRGRSSWP), similar to the P4 sequence from rice, had higher inhibitory activity with 102 μmol/mL of its IC_50_. It was also found that TIPs can be obtained from the hydrolysates of rice by-products, such as rice brans [28] and rice paste [31]. Additionally, mammalian proteins are primary materials to produce TIPs, due to their large quantity. In Table 1, TIPs of terrestrial origin can be seen. IRW and GYSLGNWVCAAK from egg white with anti-tyrosinase activity were identified [5,32]. The most studied peptides like MHIR, MYSLAMAA were derived from milk proteins, such as αS-casein, κ-casein, and β-lactoglobulin [21]. Addar et al. [17] hydrolyzed αS-casein isolated from camel milk, and found that the fraction with low molecular weight (<0 kDa) not only exhibited the highest anti-oxidative activity, but also strongly inhibited 51.21% of tyrosinase activity at a concentration of 0.2 mg/mL. At the same time, researchers also looked at the usefulness of other animal proteins with potential activities. Pongkai et al. [24] reported that protein hydrolysates from chicken feather meal, containing cysteine disulfide bonds, exhibited strong tyrosinase inhibition activity for both monophenolase (IC_50_ = 5.780 µg/mL) and diphenolase activities (IC_50_ = 0.040 µg/mL).

Although terrestrial plant protein is a common source of TIPs, studies have found that some natural plant ingredients exhibit higher anti-tyrosinase activity with lower concentration. Furthermore, terrestrial animal proteins are no longer popular in health care products and cosmetics due to religious beliefs, the risk of zoonotic disease transmission and other factors. The market needs new TIPs with better activity and safety.

### 2.2. Anti-Tyrosinase Peptides of Aquatic Origin

Oceans cover more than 70% of the earth’s surface, and aquatic species account for about half of the total global biodiversity. With the rise of blue resources, researchers have begun to explore more nutritional values and bioactive activities of aquatic organisms. Collagen is a kind of aquatic protein, which plays a vital role in several organs of the body, particularly in the bone, skin and cartilage. Aquatic collagen peptides were verified to show significant effects in human skin, such as anti-oxidation, anti-photoaging, and moisturizing, etc. [33,34]. In recent years, they have been found to reduce melanogenesis through inhibiting tyrosinase activity [35]. Generally, collagen hydrolysates with low molecular weight display better dispersion and higher hydrophobicity, thus exhibiting better bioactivity [36]. It was proved that the presence of hydrophobic amino acids at the beginning and end of the peptide chain formed extra interactions with copper active sites of tyrosinase [37]. Wang et al. [35] reported a low-molecular-weight (700–1700 Da) gelatin hydrolysate isolated from the sea cucumber wall with 55.7% of hydrophobic amino acids. The isolated peptides exhibited excellent inhibitory characteristics against tyrosinase activity and melanin synthesis in B16 cells. In contrast, Park et al. [26] gave the opposite result. They found Thunnus obesus collagen hydrolysate fractions with large molecular (>10,000 Da) weight exhibited higher anti-tyrosinase activity than those with small molecular weights (500–10,000 Da). To a great extent, the bioactivity of TIPs depends on the composition, quantity and position of characteristic amino acids [38,39,40].

It was estimated that fish waste, such as fish scales, skin and bones, accounted for approximately 60–75% of total fish weight and crude protein levels of aquatic waste were 8–35% [32]. The use of these discarded parts may be conducive to a circular economy. Therefore, waste hydrolysates have gained much attention as potential materials for TIPs. Fish by-products like grass carp scales [13], milk fish scales [25], thunnus obesus skin [26] and tuna backbone [32] have been hydrolyzed to obtain active TIPs. A modified peptide CNGVQPK derived from crocodile blood was verified to reduce melanin content in B16F1 cells through significantly inhibiting tyrosinase activity, and showed no damage to cell proliferation in human skin keratinocytes [41].

Compared with terrestrial protein sources, TIPs of aquatic origin normally have shorter peptide chains, which is beneficial for skin penetration and intestinal digestion and absorption. On account of their special ecological environment, the denatured temperature of TIPs from aquatic origin is low, which does not harm cell proliferation. Furthermore, TIPs of aquatic origin have higher solubility so as to simplify the technological process. To sum up, it is speculated that aquatic biological proteins could replace terrestrial biological proteins as the main sources for preparing active peptides, especially TIPs, in future years.

**Table 1:** The sources of food-derived tyrosinase inhibitory peptides (TIPs) and their activity.

Origin	Source	Peptides Sequences or Hydrolysates	Molecular Weight (Da)	Acitivity Evaluation	References
Terrestrial origin	Potato	Solunum tuberosum peels hydrolysates	485,980	990.44 μg KE/μg peptides	[22]
	Vicia faba pods	Broad bean pods hydrolysates	26,102	135.80 μg KE/μg peptides	[23]
	Chinese quince seeds	RHAKF	658	IC_50_: 0.93 mg/mL	[15]
	Defatted walnut meal	FPY	425	IC_50_: 1.11 mmol/L	[42]
	Liquid rice starch	LQPSHY	744	IC_50_: 0.16 mmol/L	[28]
	Rice starch	Strain hydrolysates	<1000	107.70 mgKAeq/g	[31]
	Chicken feather meal	Proteolysates	<3000	IC_50_: 0.04 µg/mL	[24]
	Egg white	IRW	340	IC_50_: 2.90 mmol/L	[32]
	Egg white	GYSLGNWVCAAK	1268	IC_50_: 3.04 mmol/L	[5]
	Milk	MHIR	555.30	IC_50_: 0.08 mmol/L	[21]
	Camel milk	αS-casein hydrolysates	>10,000	0.2 mg/mL (peptides): 39.26%	[17]
	Ganoderma lucidum	VLT	639	5.0 mg/mL (peptides): 16.00%	[43]
	Porcine skin	Proteolysates	<3000	5.0 mg/mL (peptides): 69.80%	[44]
	Chia seeds	Proteolysates	<3000	IC_50_: 0.66 mg/mL	[45]
	Sorghum grain kafirins	Proteolysates	<1000	Peptides solution: 14.20%	[46]
Aquatic origin	Rhopilema hispidum	Collagen hydrolysates	<10,000	Collagen solution: 64.00%	[47]
	Sea cucumber	Body wall gelatin	700–1700	0.1 mg/mL (peptides): 30.80%	[35]
	Grass carp fish	FTGML	567	IC_50_: 1.89 mmol/L	[13]
	Mackerel meat	VWWW	680	IC_50_: 1.25 mmol/L	[32]
	Tuna (backbone protein)	VKAGFAWTANQQLS	1519	IC_50_: 0.60 mmol/L	[32]
	Milk fish scale	MSCP	/	IC_50_: 0.75 mg/mL	[25]
	Bigeye tuna and thunnus obesus skin	Proteolysates	50,000–100,000; <1000	5.0 mg/mL: 63.10% (50,000–100,000); 56.10% (<1000)	[26]
	Zebrafish	Phosvitin-derived peptide Pt5	/	0.1 mg/mL (peptides): 16.00%	[48]

## 3. The Preparation of Anti-Tyrosinase Peptides from Food Protein

The methods for TIPs preparation include enzymatic hydrolysis, chemical hydrolysis, microbial fermentation and chemical synthesis, among which the enzymatic hydrolysis and solid phase synthesis are the two more common technologies.

### 3.1. Enzymatic Hydrolysis

Enzymatic hydrolysis has become one of the most common methods for preparing TIPs in recent years, due to mild reaction conditions and ease of process control. A variety of commercial enzymes are currently used for TIPs production, including flavourzyme, alkaline protease, neutral protease, trypsin, chymotrypsin, papain, etc. On the one hand, different enzymes have different hydrolytic effects on the same material due to the binding specificity between enzyme and substrate. On the other hand, the enzymolytic effect of the same enzyme on different raw materials is different. El-sayed used the immobilized lettuce protease to hydrolyze potato peels [22] and broad bean pods [23] respectively, and found that the tyrosinase inhibitory activity of broad bean pods hydrolysate was better than that of potato peels.

It is of importance to correctly select raw materials and proteases before enzymatic hydrolysis. In the previous discussion, lactoproteins, such as milk [17,21] and eggs [32,37], are good materials to prepare TIPs. In addition, researchers found that trypsin and chymotrypsin had specific cleavage characteristics of amino acids that contribute to tyrosinase inhibition, such as Arg, Lys, Phe, Leu, Tyr, etc. For example, trypsin as an endopeptidase cleaves Arg and Lys at the C-terminal of peptide chains, and chymotrypsin specifically cleaves Phe, Leu, Tyr, Met and Try at the C-terminal of peptide chains. Addar et al. [17] demonstrated that chymotrypsin could produce hydrophobic aromatic amino acids from αS-casein. Yap et al. [14] found that the egg albumin hydrolysate with the highest monophenolase inhibition was produced by the complex of 55% trypsin + 45% chymotrypsin. Protein materials with tyrosinase inhibitory activity usually had high contents of hydrophobic amino acids (Trp, Phe, Gly, Val, Leu, Ile, Ala, Pro and Met) and aromatic amino acids (Tyr, Trp and Phe). Hydrophobic amino acids react with other residues, free radicals or metal ions as hydrogen donor while aromatic amino acids have conjugated planar rings, which can not only absorb the ultraviolet rays, but also form π-π interactions with Cu^2+^ of tyrosinase. The conjugation with Cu^2+^ can interrupt the oxidative action of tyrosinase, thereby inhibiting the synthesis of melanin. In short, the common method to obtain TIPs at present includes enzyme species screening and enzymolytic process optimization, accompanied by effective purification.

### 3.2. Solid Phase Synthesis

Chemical synthesis of TIPs includes solid phase synthesis and liquid phase synthesis, among which the former has been developed since the 1960s. Solid phase synthesis to synthesize TIPs uses the continuous reaction of amino acids on insoluble porous carriers. It is mainly divided into 9-fluorene methoxy-carbonyl (Fmoc) synthesis and tert-butyl-carbon (Boc) synthesis according to the different protective groups added at the N-terminal and side chains of the peptide sequence [38]. Compared with Boc synthesis, The protective groups by Fmoc synthesis have the advantage of being stable in an acidic medium and being removed easily in basic solution, making Fmoc synthesis more popular in TIPs production. Ookubo et al. [49] synthesized an octapeptide LILVLLAI by Fmoc synthesis and found it could enter B164A5 cells through the skin delivery system and significantly inhibit melanin production. Kim et al. [50] established a kojic acid-tripeptide library (KO-X1X2X3) by Fmoc synthesis and verified that the tyrosinase inhibitory activity of convergence was greatly enhanced. Compared with liquid phase synthesis, solid-phase synthesis omits the purification step and overcomes the difficulty in dissolving long-chain TIPs in solution. However, it is still unsatisfactory to achieve in large-scale production, due to its complex operation and high cost.

Enzymatic hydrolysis is limited for its low yield of target peptides as a result of protease choice blindness, and solid phase synthesis is limited for its high cost. Virtual enzymatic hydrolysis on a mass data base has developed well in recent years for it can predict the sequence fragments and their corresponding bioactivities hydrolyzed by one or more specific protease(s). Virtual enzymatic hydrolysis would help experimental work go further by improving the probability of each peptide site being cleaved and difference in cleaved sites.

## 4. The Possible Hypopigmentation Mechanisms of Anti-Tyrosinase Peptides from Food Proteins

### 4.1. Mechanism of Anti-Tyrosinase Peptides by Anti-Oxidation

Melanogenesis is attributed to many factors such as UVR, alcohol, emotion, diet, nicotine and so on, among which UVR is the main reason. The synthesis of melanin including pheomelanin and eumelanin is initiated with the oxidation of L-tyrosine (L-tyr) and/or L-dihydroxyphenylalanine (L-dopa) to dopaquinone (DQ) catalyzed by tyrosinase and then DQ forms melanin through a radical-coupling pathway. They are two rate-determining steps for the production of melanin [51]. Eumelanin is responsible for brown and black pigmentation and confers a high degree of protection against UVR. Compared with eumelanin, pheomelanin is readily photodegraded and is thought to contribute to UVR damage [52]. UVB causes oxidative stress and affects the skin’s susceptibility to oxidative damage, while UVA directly induces structural DNA damage [46].

**Figure 1:**
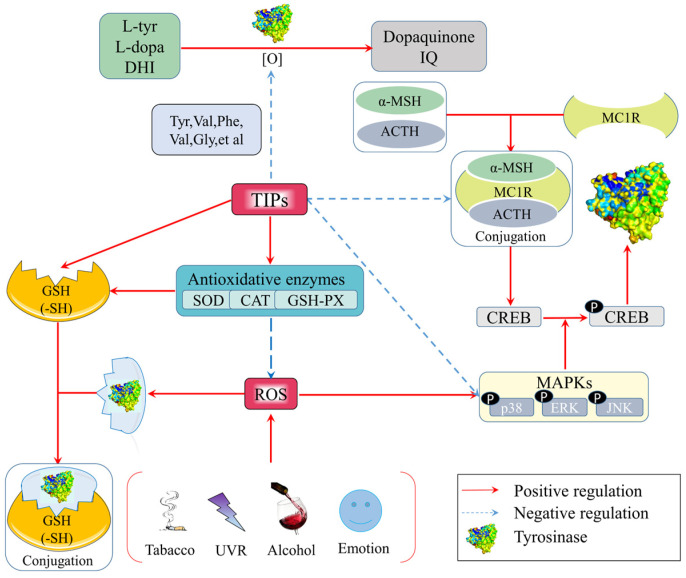
Anti-tyrosinase mechanisms of food-derived tyrosinase inhibitory peptides (TIPs) by anti-oxidation.

Generally speaking, melanogenesis is essential to skin as a barrier to absorb UVR, aiming to protect skin from sunburn. In fact, the melanocytes are under continuous low-grade oxidative stress. However, excessive stimulation of UVR leads to advanced oxidative stress and the production of free radicals, such as hydroxyl radicals, super-oxide anion radicals, hydrogen peroxides, oxygen singlets, hypochlorites, NO radicals and peroxynitrite radicals [53,54], among which super-oxides and hydroxyl radicals are the most active [35]. Free radicals are highly unstable and rapidly react with nearby biomacromolecules, resulting in a series of body sicknesses, such as DNA damage [52], cancer development and inflammatory reaction [55]. In addition, they can also cause abnormal melanogenesis as a result of tyrosinase activation. In summary, there are three ways for TIPs to inhibit tyrosinase through the photoaging system; (1) to directly scavenge free radicals as antioxidants; (2) to activate the activity of anti-oxidative enzymes such as SOD, CAT, and GSH-Px to indirectly scavenge free radicals; (3) to regulate relative gene pathways to decrease the content of tyrosinase. Related anti-tyrosinase mechanisms of TIPs through anti-oxidation are shown in Figure 1.

Previous studies reported that some skin whitening components post anti-melanogenic effects through anti-oxidative activity [17]. It seemed that peptides with strong anti-oxidative activity also showed strong anti-tyrosinase activity, suggesting a relationship between them. One of the reasonable reasons may be amino acid residues or chain fractions in both activities. Anti-oxidative amino acids compete for oxygen atoms or free radicals with L-tyr, L-dopa and 5,6-dihydroxyindole (DHI) as substrates during the synthesis reaction of melanin, thereby interrupting oxidation catalyzed by tyrosinase. On the one hand, hydrophobic amino acids, such as Val, Ala, Gly, Iso, Leu, Phe, and Pro, were found to be abundant in bioactive peptides with the capacity of scavenging free radicals [26]. Likewise, these amino acids are also important in TIPs [55]. On the other hand, aromatic amino acids (Trp, Leu, Phe, Tyr, Val, Ile) make active oxygen stable through direct electron transfer [32]. Interestingly, many studies reported that Tyr, Phe, and Val could remarkably enhance the anti-tyrosinase activity of TIPs [39,41,56]. In addition, peptides tightly binding to tyrosinase contain usually more than one Arg/Phe and have stronger inhibitory activity when combined with Val, Ala and Leu [57], while Leu and Val commonly exist in anti-oxidative peptides with Pro [53].

To resist ROS damage and protect cells from oxidative stress, the skin is well equipped with anti-oxidant defense enzyme systems, including super-oxide dismutase (SOD), catalase (CAT) and glutathione peroxidase (GSH-Px), among which SOD is vital to activate CAT and GSH-Px. TIPs can increase the activity of SOD, CAT and GSH-Px to inhibit tyrosinase that is sensitive to ROS. Castro-Jάcome et al. [46] added crude alcalase hydrolysates derived from white sorghum grain kafirins to organotypic cultures of human skin exposed to UVB. The results revealed that the treatment with hydrolysates significantly reduced the damage caused by UVB by attenuating the depletion of the activities of SOD and GSH-Px, as well as by maintaining or increasing the activity of CAT and showed a significant tyrosinase inhibition of 14.2%. Similarly, an oligopeptide FTGML derived from grass carp scales increased the activities of SOD, CAT, and GSH-Px by 63.10%, 64.53%, and 69.29%, respectively, after FTGML treatment in B16F10 cells. In addition, FTGML also promoted the GSH level in cells, thus decreasing the tyrosinase activity by 56.13% when FTGML concentration was 1.6 mg/mL [58], indicating that GSH influenced melanin production. In fact, GSH had previously been found to directly conjugate the active site of tyrosinase through its sulfydryl structure, thus interrupting the catalysis of tyrosinase [59]. In the negative feedback pathway, GSH can be activated by GSH-Px. In summary, the synergy function of enzymatic anti-oxidants is an effective strategy to suppress abnormal activation of tyrosinase and hyperpigmentation.

Free radicals stimulate a synergetic effect by means of the oxidation system and pigmentation system. For example, free radicals irritate the phosphorylation of c-Jun N–terminal kinase (JNK), signal-regulated kinase (ERK) and p38 through the mitogen-activated protein kinase (MAPK) signaling pathway [55], which can further phosphorylate the cyclic response element binding protein (CREB), ultimately increasing the expression of tyrosinase. JNK and p38 are the two most significant factors in MAPK that affect melanocyte proliferation. Hu et al. [58] verified that FTGML downregulated the phosphorylation of JNK and p38, among which p-JNK/JNK and p-p38/p38 ratios of B16F10 cells were 0.65 and 0.70 after 1.6 mg/mL FTGML treatment. The results suggested that FTGML inhibited tyrosinase-mediated melanin synthesis through downregulation of the JNK and p38 signaling pathways. In addition, melanocyte stimulating hormone (α-MSH), as one of the key receptors involved in determining mammalian skin, sensitively increases expression, due to the offense of free radicals induced by UVR. Both α-MSH and adrenocorticotropic hormone (ACTH) are well known endogenous agonists of the melanocortin 1 receptor (MC1R), and ACTH promotes the conjugation between α-MSH and MC1R. MC1R is also capable of phosphorylating CREB. A study [21] reported that a tetrapeptide MHIR derived from milk suppressed phosphorylation of the CREB at Ser133 by 37% in 3 h, indicating that MHIR could interfere with α-MSH binding to MC1R, thereby inhibiting the activation of tyrosinase.

Although there is a crossover between anti-oxidation and anti-melanogenesis pathways, concrete pathways still remain unclear. Furthermore, there are few studies about the anti-oxidative mechanism of hyperpigmentation induced by other factors except UVR. The issue of how to utilize TIPs to cure hyperpigmentation effectively continues to be adequately answered.

### 4.2. Mechanism of Anti-Tyrosinase Peptides by Occupying the Bioactive Site of Tyrosinase

Tyrosinase is a H2L2 tetramer with copper Ⅱ ions (Cu^2+^) in the active center of H subunit, which is the tyrosinase domain, and has a molecular weight of 43 kDa [60]. The tyrosinase exterior is a hydrophilic environment, but a hydrophobic cavity forms inside the active center. The active cavity contains binuclear copper ions chelated with six His residues (while CuA interacts with His61, His85 and His94 and CuB interacts with His259, His263 and His296) and a few amino acids near to the cavity entrance to induce substrates to the active site. Therefore, TIPs can slip into the active cavity and cover the catalytic site in order to inhibit the activity of tyrosinase as a disguised substrate. According to differences in amino acid composition, TIPs not only chelate with binuclear copper ions, but also form interactions with amino acid residues surrounding the active core.

#### 4.2.1. TIPs Inhibit Tyrosinase Activity by Chelating with Binuclear Copper II Ions Catalytic Core

As mentioned above, binuclear Cu^2+^ connected by an oxygen bridge play a crucial role during the catalysis of tyrosinase. Tyrosinase’s catalytic center includes different forms depending on the oxidation condition of the Cu^2+^ which contains oxygenated, deoxy, oxidized met, and half-met forms [61]. The mutual transformation of different forms is the basis of the catalytic function. Besides this, six His residues belong to part of the active site of tyrosinase and have particularly high affinity for Cu^2+^ because of their imidazole rings that can bind metals [60]. Hence, they are directly involved in the catalytic activity of the Cu^2+^. Interaction with one or more of the Cu^2+^ can properly inactivate the function of tyrosinase [62]. However, His85, His259, His263 lay near to the cavity entrance, whereas His61, His94 and His296 are located deep within the cavity. Bulky side chains of TIPs may not be favorable for forming interactions with His residues within the cavity [61]. Short sequences, such as IR, VY, LK [60], FPY [42] and ECGYF [39], were found to bind His residues nearer to the cavity entrance, thus making it easier to chelate with the Cu^2+^ catalytic core. 

**Figure 2:**
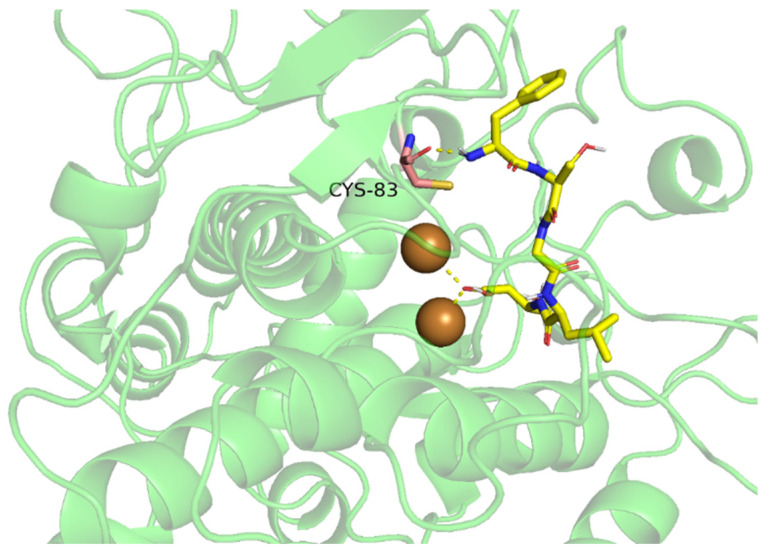
Simulated conjugation between food-derived tyrosinase inhibitory peptides (TIPs) and tyrosinase by molecular docking.

TIPs have also been simulated to have a direct interaction with Cu^2+^ [5,10]. The closer the binding between TIPs and Cu^2+^, the lower the catalytic activity of Cu^2+^. Figure 2 shows possible interactions between TIPs and tyrosinase. Functional groups, such as the imidazole ring of His [60], guanidine group of Arg [57], sulfydryl of Cys [41], aromatic nucleus of Tyr, Trp, Phe [5] and hydrazone [63], are thought to chelate with Cu^2+^ better. Nie et al. [56] made a molecular docking between heptapeptide (IQSPHFF) and tyrosinase and found that IQSPHFF could bind to Cu^2+^ and formed hydrogen bonds with five His residues at the active site. Joompang et al. [41] reported that—SH of Cys could chelate with Cu^2+^ better than —OH of Tyr, indicating that TIPs with Cys have higher anti-tyrosinase activity. Furthermore, the N–terminal penultimate Tyr of GYSLGNWVCAAK formed a covalent bond with CuB and two hydrogen bonds with His259 and His263 [5].

In silico analysis, another method of verifying TIPs’ binding to Cu^2+^ is to measure the free Cu^2+^ (CuSO_4_ as an example) chelation activity of TIPs. For example, TIPs compete with complexation indicators, such as pyrocatechol violet or tetramethyl murexide, to chelate with free Cu^2+^, thereby showing spectrophotometric change. It was reported that RHAKF’s Cu^2+^ chelation rate IC_50_ was 0.93 mg/mL, better than that of EDTA (IC_50_ = 1.13 mg/mL) [15]. In addition, stronger chelating capacity helped RHAKF inhibit tyrosinase better than NYRRE. What’s more, a peptide, FIDDDAFIR, derived from salmon by-product proteolysate, strongly chelated with Cu^2+^ of 15,309.62 µg/g protein, whose molecular weight was less than 1 kDa [64].

Even though there is a high positive correlation between Cu^2+^ chelative activity and anti-tyrosinase activity, there is a lack of conviction that TIPs inhibit tyrosinase activity by chelating with Cu^2+^ at the active site. In silico analysis simulates the most possible conjugative conformation between TIPs and tyrosinase through physical calculation, which can largely improve the credibility of TIPs’ chelating with Cu^2+^. However, none of the techniques or methods can fully prove this hypothesis.

#### 4.2.2. TIPs Inhibit Tyrosinase Activity by Binding to Amino Acid Residues of Tyrosinase Hydrophobic Cavity

According to previous studies, most of TIPs interrupted the tyrosinase function by reacting with the residues present in the cavity entrance and/or vicinity of the catalytic core but not the Cu^2+^, probably because of flexible loops, coils, and turns at the cavity entrance in contrast to the stable center [61]. During conjugation, TIPs may undergo continuous transformation, form interactions with relevant residues, and finally occupy the binding site of the substrate by the lowest energy conformation [65]. It was demonstrated that His85, His61, Phe292, Ala286, Glu256, Asn260, Val283, Phe264, Met280 and His244 made up a hydrophobic cavity and played an important role in tyrosinase catalytic activity [18]. TIPs form interaction forces, such as hydrogen bonds, hydrophobic interactions (such as π–π stacking, π–Sigma, π–Alkyl, π–Cation, Alkyl), unfavorable interactions, electrostatic interactions and Van der Waals’ forces with residues, among which hydrogen bonds and hydrophobic interactions are two main forces [13]. Table 2 shows the peptide interaction forces and residues with tyrosinase. In the molecular docking result of L-tyr, a natural substrate of tyrosinase, it was demonstrated that L-tyr interacted with His263 through π–π stacking by its benzyl ring [66]. What’s more, it also formed hydrogen bonds with Asn260, His94 and His296. Similarly, natural compounds like 4-hydroxycinnamic acid could form π–Sigma and π–π stacking interactions with Val283 and His263 through the benzyl ring [18]. According to literature overviews, ligands have some structural properties in common. For example, they have at least one aromatic ring with a phenolic group [67] and one pyranoid ring [62]. The aromatic ring of the ligands could be almost parallel to the imidazole ring of His residues through π–π interactions or other hydrophobic forces between the electron clouds of porbitals [68], and these conjugated structures improved the binding ability of TIPs to tyrosinase.

#### 4.2.3. Emerging Bioinformatic Technologies Used in Exploring Novel TIPs

Most of the existing studies used tyrosinase derived from Agaricus bisporus as the reactive enzyme, but there are only 23% amino acid sequences in common between mushroom and human tyrosinase [39]. Researchers noticed that TIPs effectively inhibiting the action of mushroom tyrosinase are usually ineffective against human tyrosinase [69]. The structural differences between them are mainly characterized by TIPs’ molecular features. However, not enough structural or kinetic information is available for human tyrosinase as a result of insufficient amounts from natural sources or heterologous expression. Hence, mushroom tyrosinase inhibition rate is still the most intuitive indicator for screening highly active TIPs in basic research.

In recent years, bioinformatics has become more closely related to basic research. Computer virtual tools on a bioinformatics base have been applied to seek new TIPs, including activity prediction, mass screening, molecular docking and molecular dynamics.

BIOPEP [70], an online bioinformatic database and analytic tool, can be used to explore new bioactive peptides according to its available protein database or your own sequences. Various activities such as anti-oxidant, anti-bacterial and anti-inflammatory are supported. Prediction tools can classify and predict the activity of peptides by amino acid patterns and physicochemical properties, so as to provide reasonable candidates for further experiment. Sasikarn et al. [71] used the k-nearest neighbor (kNN) algorithm to classify 133 peptides with known anti-tyrosinase properties from abalone, and employed the random forest (RF) algorithm to predict putative TIPs, with an accuracy of 97% and 99% using the two algorithms. Finally, two TIPs TASSDAWYR and SAPFMPDAFFRNV similar to known TIPs were screened out to show possible interactions with tyrosinase. At the same time, online docking tools like HELPDOCK Server [72] can be applied to mass screening, aiming to look for the TIPs with the highest activity.

In order to further explore the interaction between TIPs and tyrosinase, in silico analysis, represented by molecular docking and molecular dynamics, is widely used in scientific research. Common molecular docking software includes AutoDock, Gold glide, LibDock, Dock Vina, Affinity ligand, etc. This software can simulate the best conformation of peptides binding to tyrosinase and reflect TIPs’ inhibitory ability through binding energy. The lower the binding energy, the easier they are to conjugate with tyrosinase. In addition, the gold fitness score can also be used to characterize the binding degree between TIPs and tyrosinase, which is opposite to the binding energy. Deng et al. [15] used LibDock to simulate DYRRE whose gold fitness score was 215.3, while the other peptide RHAKF was 202.7, indicating that DYRRE bond closer to tyrosinase. Besides this, molecular docking is capable of simulating specific amino acids and binding sites between TIPs and tyrosinase, so as to assist in judging the inhibition type of TIPs [39].

Another software molecular dynamics is used to study dynamic evolution of the conjugates and analyze their conformational transition. Commonly used molecular dynamics software include Gromacs, Namd, Charmm, Amber, etc. System properties, such as root mean square deviations (RMSD), root mean square fluctuations (RMSF), radius of gyration (Rg) and solvent accessible surface area (SASA), are used to evaluate the stability and rationality of conjugated conformations obtained by molecular docking. RMSD value is used to confirm the stability of the enzyme–ligand complex compared to the free enzyme. RMSF value reflects the flexibility of the residues that may be involved in the conformational modification to ensure stable binding of the complex [12]. Rg value is calculated to characterize the tightness of the protein structure, and SASA value to determine surface area of the protein in contact with the solvent molecule and to characterize the hydrophobicity of the protein [1]. Molecular dynamics has been well developed in chemical tyrosinase inhibitors but hardly applied to TIPs.

### 4.3. Mechanism of TIPs by Regulating Related Gene Expression

**Figure 3:**
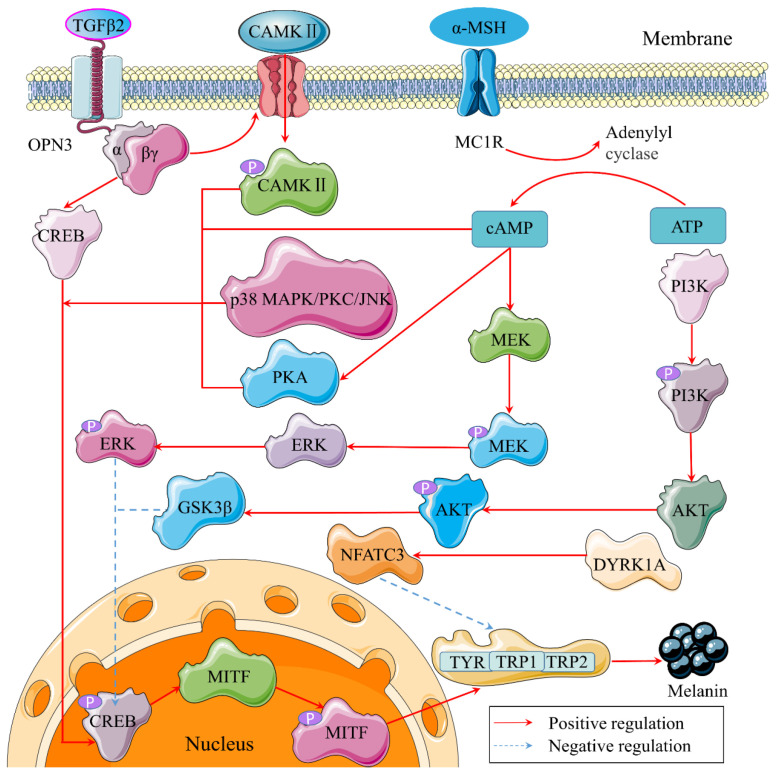
Possible tyrosinase signaling pathways in melanocytes. P is a symble of phosphoryla tion. α and βγ belong to the subunits of G-protein coupled receptors (GPCRs).

In addition to tyrosinase inhibition activity, TIPs can also reduce tyrosinase expression through related signaling pathways. Identification of critical enzymes, mediators, and signaling pathways is crucial for the development of pigmentary disorders. It is important to note that TIPs can reduce tyrosinase synthesis by mediating the anti-oxidant signaling pathway induced by photoaging, described in the above section. This part of the overview focuses on the discovered signaling pathways directly or indirectly related to melanogenesis, and not only limited to TIPs. Possible mechanisms mentioned are shown in Figure 3.

#### 4.3.1. CAMP/PKA/CREB Signaling Pathway

TIPs can reduce melanin synthesis by inhibiting gene expression of tyrosinase and its upstream factors. There are many signaling pathways involved in melanin synthesis, among which the cAMP/PKA/CREB pathway is the most critical. In this pathway, keratinocytes secrete specific cytokines, such as α-melanocyte stimulating hormone(α-MSH), and the binding of α-MSH to MC1R stimulates adenylyl cyclase (AC), thus leading to an increase in the intracellular concentration of secondary messenger cyclic adenosine monophosphate (cAMP) [51]. Subsequently, cAMP stimulates protein kinase A (PKA) translocation in the nucleus and it phosphorylates CREB at Ser133, ultimately activating CREB-mediated transcriptional activity [73]. As a transcription factor, CREB phosphorylation increases the expression level of microphthalmia-associated transcription factor (MITF), which activates tyrosinase, tyrosinase-related protein-1 (TRP-1) or tyrosinase-related protein-2 (TRP-2) by binding with the M-box or E-box consensus motif [73]. Research verified that melanin and protein levels of p-CREB, MITF, and tyrosinase were remarkably reduced by treatment with H89, a well-known PKA inhibitor [74,75], suggesting that CREB phosphorylation by PKA is a main axis for regulation of MITF transcription in melanocytes [73].

#### 4.3.2. PI3K/AKT/GSK3β Signaling Pathway

The PI3K/AKT/GSK3β pathway is known as a negatively active way to regulate melanogenesis by reducing MITF expression in melanocytes [73]. CAMP inhibits phosphatidylinositol 3-kinase (PI3K) that induces the phosphorylation of protein kinase B (AKT) at Thr308 and Ser473, thereby stimulating the activity of glycogen synthase kinase 3β (GSK3β) [51]. The activation of the PI3K/AKT cascade results in phosphorylation of GSK3β at Ser9 [76], which inhibits the binding of MITF to the tyrosinase promoter region, ultimately inducing MITF to degrade [77]. GSK-3β is a kind of extensively expressed, and evolutionarily conserved, kinase and it presents a decisive role in melanogenesis in this pathway. Zhou et al. [77] demonstrated that GSK3β played a more important role in α-mangostin-regulated melanogenesis, while there was no significant change in AKT by LY294002 (PI3k inhibitor) treatment. Hence, it was reasonable to speculate that PI3K/AKT signaling was the upstream factor for GSK3β, but it was not necessarily included in the α-mangostin-induced depigmentation process. Choi et al. [76] concluded that decursin exerted anti-melanogenic effects by upregulating PI3K/AKT/ GSK3β cascades. Further, decursin showed a suppression of MITF-mediated tyrosinase, and strong anti-melanogenic effects in 3D human skin models, suggesting its applicability as a protective agent against hyperpigmentation.

#### 4.3.3. MEK/ERK/MITF Signaling Pathway

Another negative pathway for regulating melanin synthesis is called MEK/ERK/MITF signaling pathway. In general, the extracellular signal-regulated kinase (ERK) cascade plays a main role in cell growth, but it can induce MITF phosphorylation and lead to ubiquitination in B16 melanoma cells [78]. The MEK/ERK/MITF signaling pathway triggers a signal by α-MSH via the receptor MC1R. Subsequently, cAMP phosphorylates MEK are followed by the ERK phosphorylation, and p-ERK promotes degradation via the phosphorylation of Ser73 of MITF, which finally inhibits melanogenesis [79]. In addition, the specific MEK and ERK inhibitor PD98059 could enhance the effect of cAMP and promote tyrosinase activity, ultimately increasing the content of melanin in B16F10 cells. Oh et al. [80] purified a novel peptide MGRY from marine microalga and verified that MGRY demonstrated inhibitory properties against α-MSH-induced melanogenesis via tyrosinase inhibition and melanin content in B16F10 cells. Furthermore, ERK phosphorylation was significantly enhanced after the peptide treatment. Results implied that MGRY inhibited melanogenesis due to ERK phosphorylation.

#### 4.3.4. P38 MAPK/CREB/MITF Signaling Pathway

P38 mitogen-activated protein kinase (p38 MAPK), one of the members of MAPKs, results in the up-regulation of MITF and tyrosinase as positive feedback. The MAPKs family consists of ERK, JNK and p38 MAPK. JNK was also demonstrated to enhance tyrosinase activity and melanin expression [81]. Similar to MEK, cAMP activates CREB through p38 MAPK phosphorylation, and then CREB promotes MITF expression [76]. P38 MAPK specific inhibitor SB203580 was used to interrupt p38 MAPK phosphorylation, and the same effects were present in dihydromyricetin treatment [82]. Fargesin, commonly used in the treatment of allergic rhinitis, inflammation, sinusitis and headache, was found to effectively inhibit melanin production at moderate doses in mouse B16F10 cells, normal melanocyte cell lines and zebrafish [83]. What’s more, it also strongly reduced the expression of MITF and its downstream melanogenic enzymes and tyrosinase activity through both p38 MAPK/CREB/MITF and MEK/ERK/MITF signaling pathway.

In addition to the pathways mentioned above, less well studied pathways have also been present in melanocytes, such as TGFβ2/OPN3/CAMK Ⅱ and DYRK1A/NFATC3/TYR signaling pathways. The protein expression of p-CAMK Ⅱ, p-MITF, and p-CREB were increased in melanocytes after TGFβ2 treatment. TGFβ2 upregulated tyrosinase (TYR) activity and TRP-1 and TRP-2 expression in a calcium dependent G-protein coupled manner by upregulating OPN3 expression in human skin melanocytes [84]. NFATC3 suppression and DYRK1A activation increased tyrosinase expression and melanin synthesis [85].

The pathways that affect melanin production are complex and interlaced, and the specific inhibitory mechanisms of TIPs remain unclear.

## 5. Discussion

The collection of studies presented in this overview highlight the normal sources and mechanisms in melanocytes of TIPs and emerging technologic tools used in TIPs study, aiming to improve the pigmentation problem that the cosmetics industry still cannot completely solve.

The initial sources of TIPs were plants, and later animals became the main focus. With the popularity of blue economy, aquatic TIPs have become a new research hotspot. Compared with terrestrial protein sources, TIPs of aquatic origin have better physical and biological properties, which deserve further study. Whether it is terrestrial or aquatic protein, TIPs with small molecular weight (<3000 Da) seem more active. In addition, food industry by-products are also implicated in obtaining TIPs, but their anti-tyrosinase activity do not seem to be as high as that of edible proteins. It makes more commercial sense to prepare TIPs with higher activity from edible proteins than from by-products. Until now, the production of TIPs has almost always occurred through enzymatic or chemical hydrolysis, which have disadvantages of high cost and difficulty in separating products from the reaction system. Novel extraction technologies, such as microbial fermentation, pulse electric field, supercritical fluids and virtual enzyme hydrolysis, combined with conventional methods are able to keep costs lower and increase yields. Virtual enzymatic hydrolysis can predict the peptides likely to be produced after the protein has been hydrolyzed by the specific proteases in practice, and it will be widely applied in the future, employing a mathematical base as the primary means of production of TIPs.

In silico analysis plays a very important role in simulating the conjugation of enzymes and ligands, because there is no technology that can directly observe the dynamic binding between them. Many computer tools are capable of quickly screening TIPs and predicting their activities according to the established database. However, there is neither a database of anti-tyrosinase peptides nor molecular dynamics experience for TIPs. As we all know, TIPs and anti-oxidative peptides contain common characteristic amino acids. To establish a database with both activities would be convenient and beneficial for researchers to develop novel peptides for skin protection. In the final analysis, we need to use experimental instruments to characterize the combination of enzymes and ligands as much as possible, and put molecular simulation results as an auxiliary means to better judge actual molecular combination movements. The Quantitative structure-activity relationship model (QSAR) is an effective tool for predicting biological activity of compounds. Its principle is to describe the quantitative relationships between structures with similar characteristics and their biological activity, which is lacking in spatial structure investigation between receptor and ligand; molecular docking can better solve this problem. However, current studies are only using molecular docking to verify the interaction sites between receptor proteins and active peptides. Therefore, the effective integration of these two technologies can improve the accuracy of screening efficiency of active peptides. Meanwhile, the time-consuming and laborious purification process of traditional experimental methods can be avoided by making full use of structural bioinformatics technology, thus laying a theoretical foundation for efficient screening of bioactive peptides based on the structure-activity relationship.

It is well known that most tyrosinase inhibitors work through powerful anti-oxidation. However, the specific mechanism of oxidation, especially regarding photoaging and tyrosinase activity, is not fully understood. Our skin is exposed to light all the time, triggering a complex set of reactions that goes beyond just sun tanning. In addition to freckle-removing and whitening activities, TIPs are required to have more functionalities, such as moisturizing, elasticity, anti-aging and anti-inflammation, which need further investigation. The pure peptide may exhibit a higher toxicity and weaker bioactivity than the crude extracts in practical application, suggesting a non-negligible synergetic effect of multiple compounds. What’s more, chemical inhibitors usually exhibit stronger tyrosinase inhibition rather than bioactive peptides, although they pose a security risk. Therefore, the synergetic effect of various tyrosinase inhibitors and agents with different bioactivities deserves study. More importantly, future studies should consider mechanisms as to how TIPs regulate tyrosinase activity, activated by other factors, such as nicotine, alcohol, emotion, diet and endocrine function.

TIPs can inhibit tyrosinase activity by chelating with tyrosinase’s copper Ⅱ ions’ centers. Copper is an essential trace mineral in the human body, and it is associated with some pathogenesis, due to its pro-oxidation effect. Therefore, the copper chelating ability of TIPs should be emphasized and utilized. Peptides with copper chelating ability can be isolated by copper-immobilized affinity chromatography and prepared by tyrosinase inhibition activity as an evaluation index. There have been no reports about this method. It can be underscored that more research should focus on how copper ions in tyrosinase catalyze L-tyr or L-dopa and how TIPs inhibit the catalytic reaction of copper ions.

The Reconstructed human epidermal model (RHE) was developed as an alternative to animal tests to predict acute skin and eye irritation, and now is further used to verify whitening activity. On this basis, the model may be used in studying TIPs’ inhibitory pathway. In addition, the zebrafish model can also serve as an excellent model for studying the melanogenesis signaling pathway, as it has up to 75% homology with human gene sequences. Finally, cytokine inhibitors make a reverse argumentation that is more convincing. Although the signaling pathways regulating tyrosinase expression have been roughly clarified, the specific targets of TIPs in these pathways are still obscure. Most important of all, more studies should be conducted instead of just observing the expression changes of several upstream factors. Future research will need to combine genomics with proteomics and clinical trials to provide better evidence for TIP function.

## Figures and Tables

**Table 2:** Interactive forces and amino acid residues between TIPs and tyrosinase.

Peptide Sequences	Tyrosinase Inhibition Activity	Interaction Forces and Residues	References
IQSPHFF	IC_50_: 1.70 mmol/L	Hydrogen bond: Lys229, Gly250, Ser276	[56]
		π–π stacking: His266	
FTGML	IC_50_: 1.89 mmol/L	Hydrogen bond: Lys147, Trp53	[13]
		π–π stacking: Trp53	
		π–Alkyl: Ile39, Phe41	
		Attractive Charge: Asp51	
NGVQPKY	/	Hydrogen bond: Asn260, His94, His296	[41]
		π–π stacking: His263	
		π–Alkyl: Val283	
CNGVQPK	/	Hydrogen bond: Pro277, Leu275, Gly281, Gly257	[41]
		π–Alkyl: Asn260, Glu256, Met257	
IR	/	Hydrogen bond: His85, His94, Glu256, His259, Asn260, His263, Gly281, His296	[60]
		π–Alkyl: His244, His263	
		Alkyl: Val283, Ala286	
LK	/	Hydrogen bond: His61, His85, Glu256, His259, His263, Met280, His296	[60]
		π–Alkyl: His244, His263	
		π–Amide stacking: His244, His263	
		Alkyl: Val283, Ala286	
VY	/	Hydrogen bond: His85, His263, Gly281, His296	[60]
		π–π stacking: His263	
		π–Alkyl: His85	
		Alkyl: Val283	
		π–Sigma: Val283	
		π–Amide stacking: His85	
GYSLGNWVCAAK	IC_50_: 3.04 mmol/L	Hydrogen bond: Tyr65, His259, His263, Arg268, Gly281, Glu322	[5]
		Hydrophobic Interaction: Ala80, Cys83, Arg321, His85, Val283, Asn81,	
		Glu189, His244, Val248, Asn260, Phe264, Ala323	
		Covalent bond: CuB	
ECGYF	IC_50_: 0.46 mmol/L	Hydrogen bond: Met280, Tyr65, Asn260, His263	[39]
		Hydrophobic interaction: Phe264, Pro284	
FPY	IC_50_: 3.22 mmol/L	Hydrogen bond: Asn260, Pro81	[42]
		π–π stacking: Ser282, His263	
		π–Alkyl: Ala286, Val283	
		π–Sigma: Val283	
		π–Cation: His259	
RHAKF	IC_50_: 1.15 mg/mL	Hydrogen bond: Gln351, Asp336, Ile96, Gln74, His76	[15]
		Donor–Donor interaction: Gln74	
		π–Alkyl: Ile328	
		π–Cation: Lys5	

## Data Availability

Not applicable.
